# Pulmonary consequences of experimentally induced stroke: differences between global and focal cerebral ischemia

**DOI:** 10.3389/fphys.2024.1511638

**Published:** 2024-12-12

**Authors:** Petra Somogyi, Ibolya Tóth, Bence Ballók, Zaid Hammad, Ramez A. Hussein, Fruzsina Kun-Szabó, József Tolnai, Judit Danis, Szilvia Kecskés, Gergely H. Fodor, Eszter Farkas, Ferenc Peták

**Affiliations:** ^1^ Department of Medical Physics and Medical Informatics, University of Szeged, Szeged, Hungary; ^2^ Cerebral Blood Flow and Metabolism Research Group, Hungarian Centre of Excellence for Molecular Medicine–University of Szeged, Szeged, Hungary; ^3^ Department of Cell Biology and Molecular Medicine, University of Szeged, Szeged, Hungary; ^4^ Department of Immunology, University of Szeged, Szeged, Hungary

**Keywords:** cerebral ischemia, stroke, respiratory mechanics, animal model, inflammation, lung injury

## Abstract

**Introduction:**

Cerebral ischemia leads to multiple organ dysfunctions, with the lungs among the most severely affected. Although adverse pulmonary consequences contribute significantly to reduced life expectancy after stroke, the impact of global or focal cerebral ischemia on respiratory mechanical parameters remains poorly understood.

**Methods:**

Rats were randomly assigned to undergo surgery to induce permanent global cerebral ischemia (2VO) or focal cerebral ischemia (MCAO), or to receive a sham operation (SHAM). Three days later, end-expiratory lung volume, airway and respiratory tissue mechanics were measured at positive end-expiratory pressure (PEEP) levels of 0, 3 and 6 cmH_2_O. Bronchial responsiveness to methacholine, lung cytokine levels, wet-to-dry ratio, blood gas parameters and cerebral stroke markers were also evaluated.

**Results:**

Global and focal cerebral ischemia had no significant effect on end-expiratory lung volume, bronchial responsiveness, and arterial blood gas levels. No change in respiratory mechanics and inflammatory response was evident after 2VO. Conversely, MCAO decreased airway resistance at PEEP 0, deteriorated respiratory tissue damping and elastance at all PEEP levels, and elevated Hct and Hgb. MCAO also caused lung edema and augmented IL-1β and TNF-α in the lung tissue without affecting IL-6 and IL-8 levels.

**Discussion:**

Our findings suggest that global cerebral ischemia has no major pulmonary consequences. However, deteriorations in the respiratory tissue mechanics develop after permanent focal ischemia due to pulmonary edema formation, hemoconcentration and cytokine production. This respiratory mechanical defect can compromise lung distension at all PEEP levels, which warrants consideration in optimizing mechanical ventilation.

## Introduction

Stroke is the second leading cause of death and the third leading cause of disability worldwide, with ischemic cerebral stroke contributing the majority of cases (Feigin et al., 2022). Poor clinical outcome is partly due to multiple organ dysfunction (Robba et al., 2020; Qin et al., 2016). Of the peripheral organs, the lungs are among the most affected by stroke (Qin et al., 2016; Pinto et al., 1998; Shim and Wong, 2016; Liu et al., 2018; Meisel et al., 2005; Chamorro et al., 2007; Santos Samary et al., 2016).

Various mechanisms may contribute to post-stroke respiratory impairment. Ischemic stroke activates an enhanced immune response in the brain known as neuroinflammation, while it causes immunosuppression in the periphery, making the lungs susceptible to infections (Shim and Wong, 2016; Liu et al., 2018; Santos Samary et al., 2016; Dirnagl et al., 2007; Neumann et al., 2015; Matin et al., 2022). Indeed, pulmonary infections account for the vast majority of all post-stroke infections, since impairment of immune barriers in the lungs and intestines allows a variety of bacteria to translocate into the lungs (Stanley et al., 2016; Su et al., 2022; Hilker et al., 2003). Furthermore, the complex innervation of the lungs via cholinergic pathways of the vagus nerve and the non-adrenergic non-cholinergic (NANC) nervous system acts as an interface between inflammatory reactions and the nervous system through the release of various endogenous bronchoactive mediators (Fodor et al., 2014; Barnes, 1996; van der Velden and Hulsmann, 1999). This pathway is a potential source of lung injury and neurogenic pulmonary edema that develops frequently in patients with ischemic stroke (Naseh et al., 2020). This edematous response is a result of increased pulmonary capillary permeability due to the release of various inflammatory mediators from the cerebral tissue, and of pulmonary volume overload subsequent to increased systemic vascular resistance related to neural dysfunction (Zhao et al., 2020). While the exact contribution of these pathways has not been clarified, the extent and distribution of affected brain areas may influence the individual roles of these mechanisms, resulting in different adverse pulmonary consequences.

Mechanical ventilation is commonly required in patients admitted to the hospital for acute stroke (de Montmollin et al., 2020), and changes in respiratory mechanics can fundamentally determine the optimal management of this lifesaving modality. However, the effects of stroke on respiratory mechanics and function are poorly understood, with no comparison between global and focal cerebral ischemia. Therefore, we aimed to reveal differences in the respiratory effects of global and focal cerebral ischemia via comprehensive characterization of the end-expiratory lung volume, airway resistance, and respiratory tissue mechanical parameters in animal models of ischemic stroke at various levels of PEEP. We also assessed how complex lung injury subsequent to stroke affects the respiratory mechanics and gas exchange in response to alterations in the positive end-expiratory pressure (PEEP). These functional assessments were complemented by characterizing bronchial responsiveness, gas exchange outcomes, lung and brain histology, pulmonary edema, and lung inflammatory response.

## Materials and methods

### Ethical statement

This experimental protocol was approved by the National Food Chain Safety and Animal Health Directorate of Csongrád-Csanád County, Hungary (no. XXXII./834/2022) on 22 April 2022. All procedures were carried out according to the guidelines of the Scientific Committee of Animal Experimentation of the Hungarian Academy of Sciences (updated Law and Regulations on Animal Protection: 40/2013 [II. 14.], the Government of Hungary) and in compliance with the ARRIVE guidelines. The investigators understand the ethical principles under which the journal operates and that their work complies with the animal ethics checklist.

### Experimental animals and group allocations

**FIGURE 1:**
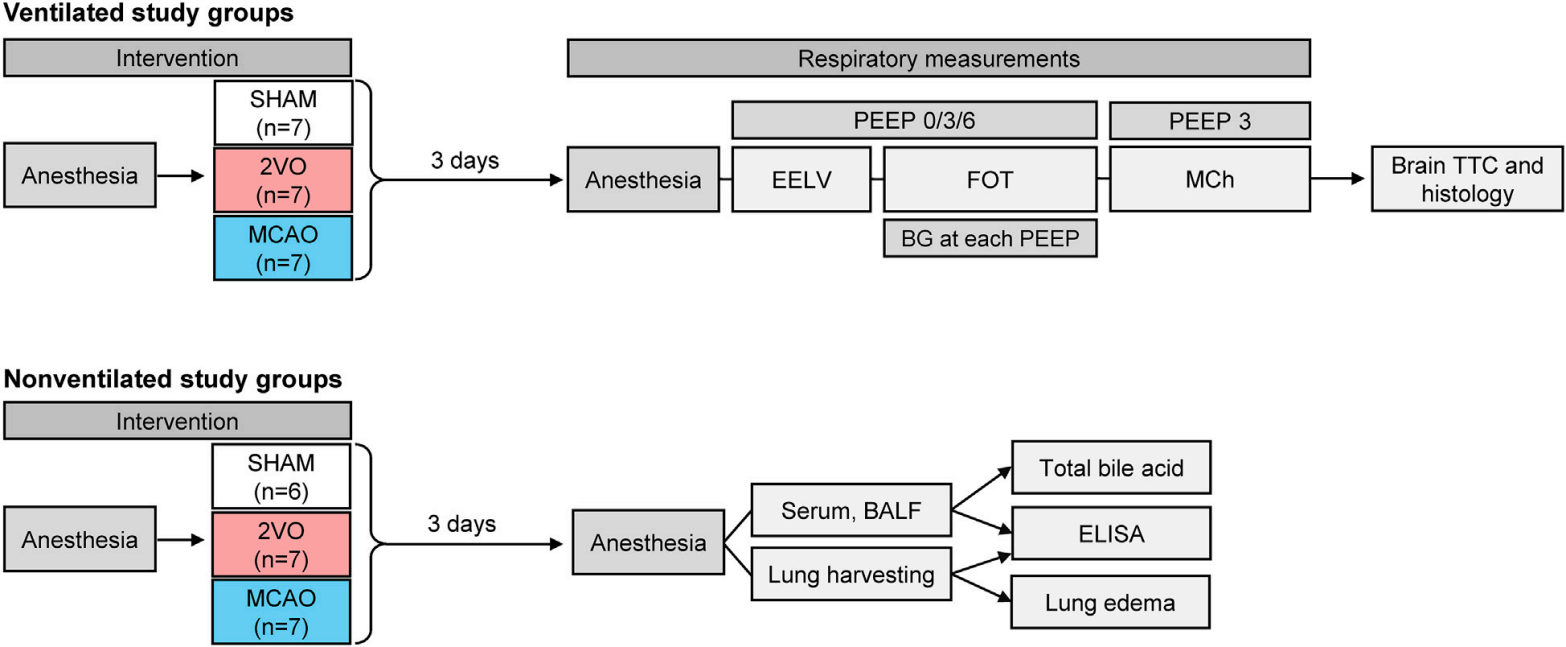
Scheme of the experimental protocol. Rats in ventilated study groups randomly underwent bilateral occlusion of the common carotid artery (Group 2VO), unilateral occlusion of the middle cerebral artery (Group MCAO), or sham operation (Group SHAM); These animals were used to assess static lung volume (EELV), forced oscillatory respiratory mechanics (FOT), methacholine responsiveness (MCh), determination of brain infarct size (TTC), and histological changes in the brain. Non-ventilated animals received the same interventions; these rats were used to measure systemic inflammatory responses from blood serum, and organ-specific inflammation in lung tissue and bronchoalveolar lavage fluid (BALF) without the confounding effect of mechanical ventilation.

Twenty-nine male Sprague-Dawley rats (Charles River Laboratories, Germany) aged 8–10 weeks (286–388 g) were included in the ventilated study groups and randomly allocated to undergo one of the following interventions based on a pre-generated random sequence by MS Excel: bilateral common carotid artery occlusion group (two-vessel occlusion; Group 2VO, N = 7), unilateral middle cerebral artery occlusion group (Group MCAO, N = 13), sham-operated control group (Group SHAM, N = 9). The animals had access to food and water *ad libitum* before the start of the experiments and during survival time and were housed according to the 3R guidelines. Since prolonged mechanical ventilation required for the measurements of respiratory function *per se* triggers lung inflammatory response (Katira, 2019), another 23 rats (2VO N = 7, MCAO N = 10, SHAM N = 6) without mechanical ventilation were allocated to investigate lung inflammatory response after the interventions. The scheme of the intervention procedure for each protocol group and the measurement protocol is outlined in Figure 1.

### Induction of cerebral ischemia

The animals were anesthetized with 4% isoflurane in a mixture of oxygen and nitrous oxide (in a 2:3 ratio) using an inhalation mask on day 0. Anesthesia was maintained with 2.5% isoflurane for the rest of the procedure. Intramuscular injection of atropine (0.05 mg/kg) was administered to prevent mucus production in the airways due to invasive procedures. Lidocaine on the shaved neck skin was applied topically to provide local anesthesia. The rats were placed on a heating pad (Homeothermic Blanket System, Harvard Apparatus, Holliston, MA, United States) to maintain body temperature around 37°C ± 0.5°C throughout the interventions.

#### 2VO group

An incision was made on the neck and the common carotid arteries were carefully separated from neighboring tissues. Surgical threads were then used to occlude both arteries (Farkas et al., 2007).

#### MCAO group

An incision was made on the neck, then the right common carotid artery, and the external and internal carotid arteries were carefully dissected. An incision was made on the common carotid artery and an intraluminal suture (type 403945PK10, Doccol Corporaion, Sharon, MA, United States) was advanced until it reached and permanently occluded the middle cerebral artery. Surgical threads were used to secure the suture in place and prevent bleeding from the arteries (Luckl et al., 2012).

#### SHAM group

In pilot experiments, there were no statistical differences between respiratory mechanics in the sham-operated animals when the surgery was in accordance with the 2VO or MCAO approach. Since MCAO is a more invasive surgery than 2VO, we chose the sham MCAO group as our SHAM group in this study. All procedures were performed as described above, except that there was no vascular occlusion in these animals.

After surgery, carprofen (5 mg/kg) was administered subcutaneously for pain management. The rats were then allowed to recover from anesthesia and housed under close observation with access to food and water *ad libitum* in the next 3 days when the experiments were performed. This time window was selected based on previous studies demonstrating that 3 days are needed for the ischemic injury and cerebral edema to fully develop in the brain (Millikan and McDowell, 1981; Castillo et al., 1998; Ropper and Shafran, 1984).

### Animal preparations for respiratory measurements

Three days after interventions, rats were anesthetized with an intraperitoneal injection of sodium pentobarbital (45 mg/kg). Tracheostomy was achieved by advancing an endotracheal tube into the trachea, which was connected to a small animal ventilator (Rodent Ventilator Model 683 Harvard Apparatus Inc.) and volume-controlled ventilation was applied (60 breaths/min, 7 mL/kg tidal volume, PEEP of 3 cmH_2_O). The femoral vein and artery were cannulated for drug administration, blood gas measurement, and blood pressure monitoring. Anesthesia was maintained with IV administration of sodium pentobarbital (12 mg/kg, every 20 min) for the rest of the experiment.

### Measurement of end-expiratory lung volume (EELV)

After completion of the animal preparation, the EELV was measured as previously described (Janosi et al., 2006). Briefly, rats were placed in an airtight Plexiglas whole-body plethysmograph, with the tracheal cannula leading through its front panel. An alveolar recruitment maneuver was applied by superimposing two inspiratory cycles to standardize the volume history. The trachea and the box were closed at end-expiration, and changes in the trachea and box pressure were recorded during spontaneous breathing efforts of the animals. EELV was calculated by applying the Boyle-Mariotte law while a PEEP of 0, 3, and 6 cmH_2_O was maintained. End-expiratory lung volume normalized to actual body mass (nEELV) was also calculated as EELV/BM.

### Measurement of respiratory mechanics

The rats were placed on a heating pad and neuromuscular blockade was achieved by IV administration of pipecuronium bromide (0.2 mg/kg, every 20 min, ARDUAN 4 mg, Richter Gedeon, Budapest, Hungary). Airway and tissue mechanics were measured using the forced oscillations technique, as previously described (Petak et al., 1997). Briefly, the tracheal cannula was connected to a loudspeaker-in-box system at end-expiration and the input impedance spectrum of the respiratory system (Zrs) was measured for 8 s. During measurements, a small-amplitude pseudorandom signal was applied to the tracheal cannula through a polyethylene wave tube (2 mm ID, 1 m length). Pressure signals were recorded at the loudspeaker and the tracheal end of the wave tube using miniature pressure transducers (model 24PCEFA6D; Honeywell, Charlotte, NC, United States), and Zrs was calculated as the load impedance of the wave tube. The mechanical properties of the respiratory system were characterized by fitting a well-validated constant-phase model to the Zrs spectra (Hantos et al., 1992). The model comprises frequency-independent airway resistance (Raw) and airway inertance (Iaw) in series with a viscoelastic constant-phase compartment that includes tissue damping (G) and tissue elastance (H). Measurements were performed at PEEP levels of 0, 3, and 6 cm H_2_O.

### Assessment of lung responsiveness

To assess lung responsiveness to exogenous constrictor stimuli, intravenous methacholine infusion (MCh) was administered in increasing doses (0, 4, 8, 16, 32 μg/kg/min) while maintaining a PEEP level of 3 cmH_2_O. Forced oscillatory measurements were performed during each infusion rate after reaching steady-state hemodynamic and respiratory conditions, as described above.

### Blood sample analysis

0.15 mL of arterial blood samples were collected at each PEEP level to assess the arterial partial pressure of oxygen (PaO_2_) and carbon dioxide (PaCO_2_), and hematocrit (Hct) and hemoglobin (Hgb) levels by using a point-of-care blood analyzer system (epoc Reader and Host, Epocal Inc., Ottawa, Canada). Due to technical reasons (blockade of the arterial line or a software update of the blood gas machine), withdrawal of blood samples was not feasible in two mechanically ventilated rats (one 2VO and one from MCAO groups).

### Evaluation of infarct size

The rats were sacrificed under deep anesthesia and the brains were harvested. To assess infarct size relative to brain size, triphenyl tetrazolium chloride (TTC) staining was used. The brains (N = 5) were cut into 2 mm-thick slices using a brain slicer matrix surrounded by dry ice. Brain slices were placed in 2% TTC solution and were incubated at 37°C for 20 min. The ischemic infarct was manually delineated, and its relative size was calculated in Nikon NIS Elements Imaging Software (Nikon Instruments Inc.).

### Evaluation of neuronal ischemic injury and glial responses

The brains obtained from the ventilated rats were embedded in paraffin and cut into 4 µm thick coronal forebrain sections and selected from two coronal planes according to the Paxinos and Watson atlas coordinates (bregma +0.7 mm, −3.14 mm). The sections were then deparaffinated in xylene and alcohol and heated in 0.01 M citrate buffer solution at 95°C for 20 min. Nonspecific protein binding sites were blocked with 10% normal goat serum (Merck, Kenilworth, United States). The presence or absence of neuron processes was assessed with microtubule-associated protein 2 (MAP-2) (rabbit, 1:1,000 dilution, catalog no. AB5622, Sigma Aldrich, Merck, Darmstadt, Germany), astrocytes were labeled with the glial fibrillary acidic protein (GFAP) astrocyte marker (mouse, 1:1,500, 4°C O/N, cat. no. G3893, Sigma Aldrich, Merck, Darmstadt, Germany), while microglial activation was identified with ionized calcium-binding adapter protein (Iba-1) (rabbit, 1:300, 4°C O/N, cat. no. ab153696, Abcam Plc., Cambridge, United Kingdom). To label astrocytes with green, Goat anti-Mouse Alexa Fluor IgG 488 (Thermo Fisher, United States, A-11001, 1:1,000, 2 h) was used, and Goat anti-Rabbit Alexa Fluor IgG 568 (Thermo Fisher, United States, A-11036, 1:1,000, 2 h) was used to label MAP-2 and Iba-1 with a red fluorescent signal. Slices were mounted and cover slipped with Fluoromount G (Thermo Fisher, United States, 00-4,959-52) containing DAPI. Using a fluorescent light microscope (Leica DM LB2, Leica Microsystems Wetzlar GmbH, Wetzlar Germany), microscopic images were taken with a LEICA DFC250 camera (Leica Microsystems Wetzlar GmbH, Wetzlar Germany), then analyzed with ImageJ software (National Institutes of Health, Bethesda, Maryland, United States). The experiments were repeated on three slides per plane and per rat, and the results were averaged for each region of each animal. The potential neuronal process and astrocytic loss were characterized by the estimation of the relative surface area covered by immunopositive MAP-2 or GFAP cells. In Iba1-immunolabeled slices, microglia activation was characterized by a ramification index as previously reported (Toth et al., 2020). Lower ramification index value indicates more activated microglia.

### Measurement of inflammatory responses in the lungs, blood serum samples and bronchoalveolar lavage fluid (BALF)

To exclude the potential biasing effects of prolonged mechanical ventilation on cytokine assays and lung interstitial edema development, separate groups of rats received the same MCAO (N = 7), 2VO (N = 7) and SHAM (N = 6) surgeries and were housed as described above.

Rats were anesthetized, tracheostomized and the femoral arteries were prepared as described above. 2 mL of arterial blood was collected for immunological analysis and was centrifuged at 3,000 rpm for 10 min, to obtain serum samples. Bronchoalveolar lavage was performed by introducing 2 mL of warm normal saline into the left lung and withdrawn by gentle suction to collect BALF samples. The BALF samples were centrifuged at 3,000 rpm for 10 min. Serum and BALF supernatant were snap-frozen and held at −80°C until use.

For protein isolation, fresh lung tissue samples were weighed, cut into small pieces, and ice-cold lysis buffer supplemented with 0.5% SDS and 1% Halt™ Protease and Phosphatase Inhibitor Cocktail (Thermo Scientific) was added (9 μL/mg tissue). Samples were homogenized in lysis buffer with an Ika Ultra Turrax T8 tissue homogenizer, centrifuged at 16 000 g, 4°C for 15 min to pellet debris and lysates were transferred to a new tube. The samples were snap-frozen and stored at −80°C until use.

Tissue lysates, serum samples and lavage samples were used to determine interleukin (IL)-1β, IL-6, IL-8 and tumor necrosis factor (TNF)-α levels by enzyme-linked immunosorbent assay (ELISA) according to the manufacturers’ instructions (Rat IL-1β Standard ABTS ELISA Development Kit, Rat IL-6 Standard ABTS ELISA Development Kit, Rat TNF-α Standard ABTS ELISA Development Kit ELISA, Peprotech; Rat IL-8 ELISA Kit, ABClonal).

### Evaluation of pulmonary edema

To assess the presence of pulmonary edema, the wet-to-dry ratio and perivascular edema of the lung was measured. For the wet-to-dry ratio, the superior lobe of the right lung was cut and kept in an oven at 60°C for 24 h. The weight of the tissues was measured before and after the drying procedure. Since probably dehydration played a major role in body mass loss during the 3-day intervention period, the wet-to-dry ratio was calculated as wet tissue weight (mg) x 100 - tissue weight loss (mg)/body mass (g).

Tissue samples from the left lung were embedded in paraffin, and 7-µm-thick sections were stained with hematoxylin and eosin, as detailed previously (Schranc et al., 2022). The total edematous area around the vessel and the area of the vessel was manually delineated using the Nikon NIS Elements Imaging Software (Nikon Instruments Inc.), and the pulmonary edema index was calculated as follows: [total area – vessel area] / [vessel area].

### Assessment of aspiration from bile acid measurements

The potential occurrence of pulmonary aspiration of gastric contents in the animals of the non-ventilated groups was assessed by measuring bile acid in the BALF. This was performed by using a *Total bile acids 21 FS* kit, according to the manufacturer’s instructions (catalog no. 1 2238 99 10,930, Diagnostic Systems, Holzheim, Germany). The measurement limit of this assay is 2 μmol/L.

### Statistical analysis

Data are expressed as mean ± standard deviation (SD). The Shapiro-Wilk test was used to test the normality of the data distribution. Two-way repeated measures ANOVA with Holm-Sidak *post hoc* test was used to evaluate data on end-expiratory lung volume, respiratory mechanics, MCh provocation, and blood gas. One-way ANOVA was used to evaluate data on cerebral infarct size and lung tissue weight loss. Pearson’s correlation was used to evaluate the correlations between Hct/Hgb and body mass loss. A sample size estimation for two-way repeated measures ANOVA, where the respiratory tissue elastance (H) was considered as the primary variable, with a power of 0.8 and an alpha of 0.05, indicated that at least 7 animals are required to detect a statistical difference of at least 20% between group means. Statistical tests were performed with a significance level of p < 0.05 (SigmaPlot, version 14, Jandel, Inc., United States).

## Results

### Study population

In the ventilated study group, four of the 13 rats initially treated in the MCAO group did not survive the MCAO intervention due to acute lethal consequences of the intervention, and two more were excluded due to the lack of cerebral ischemia, as evidenced by TTC staining, leaving seven rats in the final study group. In the SHAM group, one rat was excluded due to the development of pneumothorax during mechanical ventilation and one rat due to lethal respiratory complications after sham surgery, leaving seven animals in the final SHAM study group. All rats in the 2VO group survived the intervention and were included in the final analyzes. In the non-ventilated study group, 4 of the 10 rats in the MCAO group died following the induction of focal ischemia, leaving six animals in the final study.

### Validating the cerebral ischemia model and its pathophysiological effects

Since no difference was observed in body masses of the ventilated and nonventilated study groups, changes in body mass were evaluated on pooled data. No significant changes in body mass were observed in rats in the SHAM group (350 ± 13 g vs. 345 ± 22 g; day 0 vs. day 3, p = 0.854) during the 3-day observation period, and rats in the 2VO group showed slight but significant weight loss (333 ± 22 g vs. 320 ± 24 g, day 0 vs. day 3, p = 0.04). Similar to previous reports (Desgeorges et al., 2017; Dittmar et al., 2003), MCAO resulted in significant loss of body mass (349 ± 22 g vs. 260 ± 21 g, day 0 vs. day 3, p < 0.001).

**FIGURE 2:**
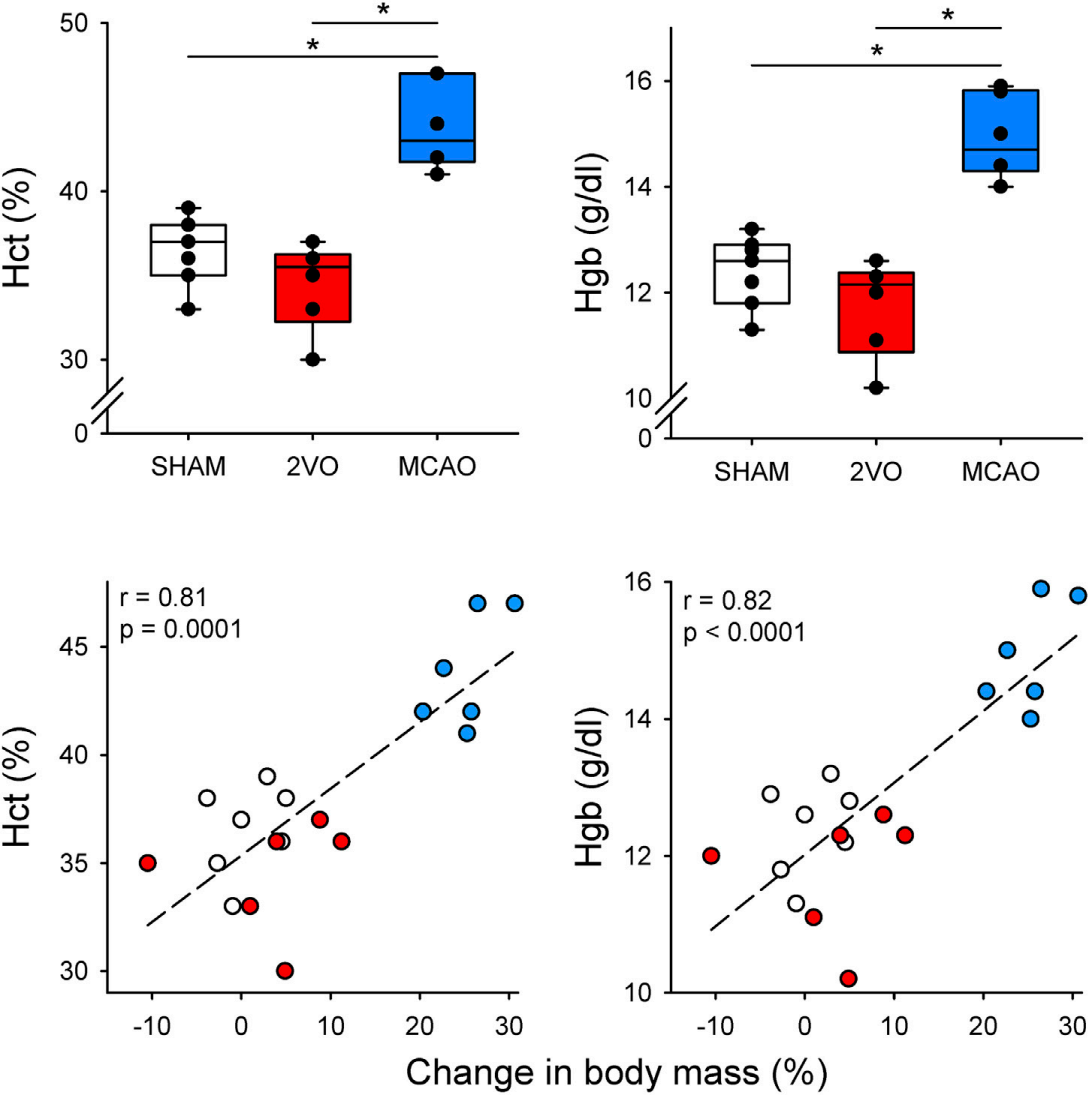
Blood hematocrit (Hct) and hemoglobin (Hgb) and their correlation with changes in body mass on day 3 in sham-operated animals (SHAM, N = 7), and after bilateral occlusion of the common carotid artery (2VO, N = 6) and occlusion of the middle cerebral artery (MCAO, N = 6). Dashed lines: linear regression. *: p < 0.001.

The results obtained from the arterial blood sample analyzes are shown in Table 1 and Figure 2. Neither 2VO nor MCAO caused significant alterations in PaCO_2_ and PaO_2_ levels (Table 1). However, the Hct and Hgb values obtained in the rats in the MCAO group were significantly higher compared to the 2VO and SHAM groups (p < 0.001 for both, Figure 2.). Examination of the correlations between the elevations of Hct and Hgb and the change in body mass revealed a strong and significant correlation (r = 0.80 and r = 0.81 for Hct and Hgb, respectively, p < 0.0001 for both, Figure 2.).

**TABLE 1:** Arterial blood gas analysis to show arterial partial pressure of CO_2_ (PaCO_2_) and O_2_ (PaO_2_) obtained at PEEP levels of 0, 3, and 6 cmH_2_O.

	PaCO_2_ (mmHg)			PaO_2_ (mmHg)		
	PEEP0	PEEP3	PEEP6	PEEP0	PEEP3	PEEP6
SHAM	35.8 (3.6)	34.7 (3.0)	36.7 (2.6)	86.9 (19.5)	97.7 (12.3)	101.6 (11.6)
2VO	37.0 (4.8)	35.8 (4.9)	37.1 (5.7)	75.9 (31.1)	83.0 (18.3)	86.8 (17.8)
MCAO	34.5 (2.8)	36.4 (8.9)	37.3 (6.8)	91.6 (29.5)	89.5 (30.8)	108.5 (7.4)

Abbreviations: PEEP, positive end-expiratory pressure; SHAM, sham surgery; 2VO, bilateral common carotid artery occlusion; MCAO, middle cerebral artery occlusion. Data are given as mean (SD).

**FIGURE 3:**
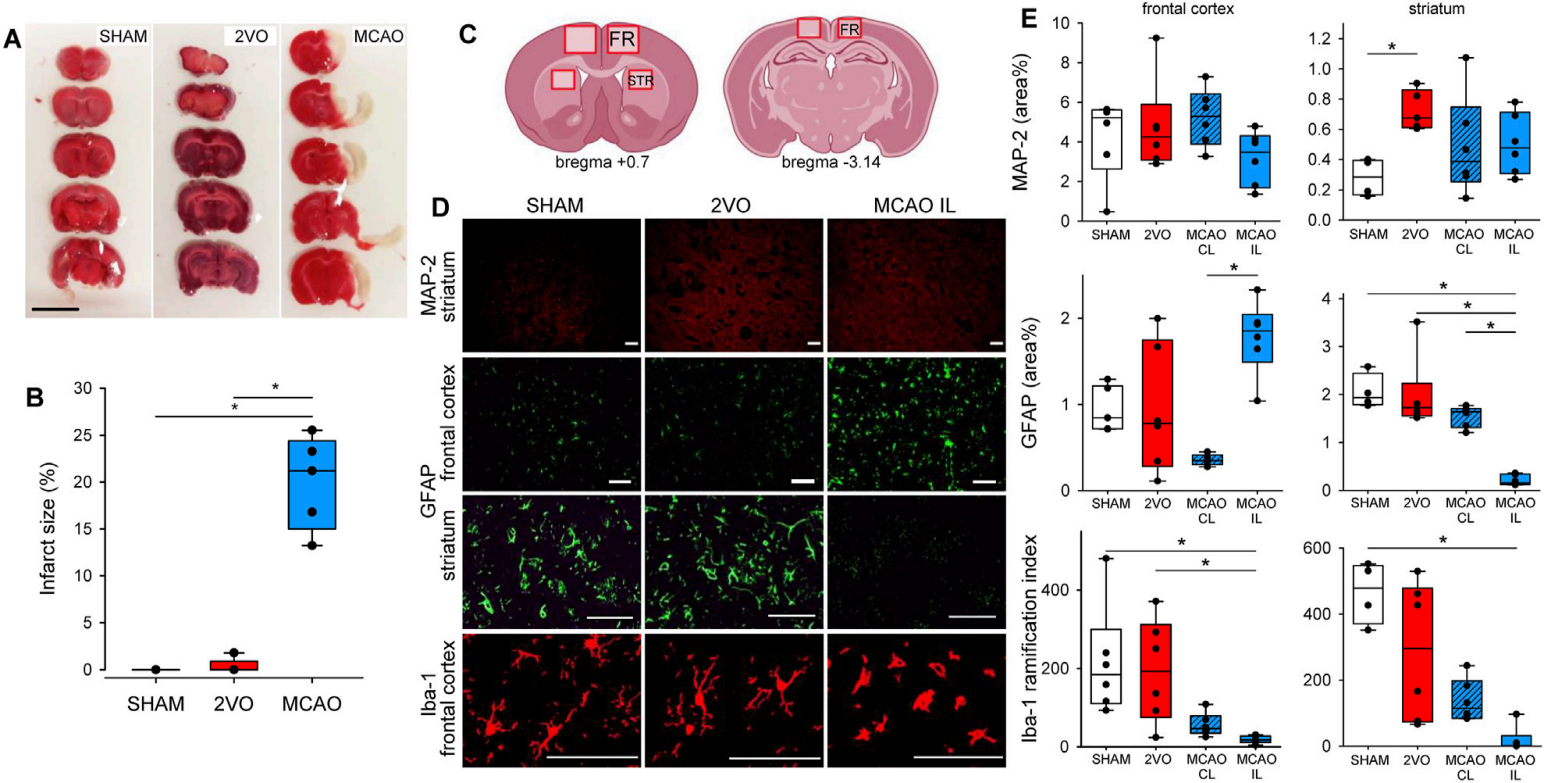
Confirmation of ischemic injury caused by cerebrovascular occlusions. Panel **(A)** representative images of brain slices stained with triphenyl-tetrazolium chloride (TTC) demonstrating ischemic infarcts (unstained area). Scale bar = 1 cm. Panel **(B)** Estimation of relative infarct size. N = 5 for each group. Panel **(C)** schematic representation of the brain sections indicating the regions of interest for the quantification of immunolabeling. Panel **(D)** representative images of brain sections immunostained for MAP-2, GFAP, and Iba-1 from the frontal cortex and the striatum. Scale bar = 100 µm. Panel **(E)** quantification of the relative surface covered by the immunolabeling of MAP-2 and GFAP, and the ramification index of Iba-1 positive microglia of each region of the brain. N = 6 for each group. SHAM: sham surgery, 2VO: bilateral common carotid artery occlusion, MCAO: middle cerebral artery occlusion; MCAO CL: contralateral hemisphere to MCAO; MCAO IL: ipsilateral hemisphere to MCAO; FR, frontal cortex; STR, striatum; MAP-2, microtubule-associated protein 2; GFAP, glial fibrillar acidic protein; Iba-1, ionized calcium-binding adapter molecule 1. *: p < 0.05.

Histological analysis of brain slices obtained from the ventilated protocol was performed to confirm and evaluate the degree of cerebral ischemic injury (Figure 3). A clear focal infarct located in the frontal and temporal cerebral cortex and striatum was obvious 3 days after MCAO in TTC stained sections (Figures 3A,B). In contrast, no mature infarct was detected in the 2VO or SHAM groups (Figures 3A,B). Immunostaining targeting neurons (MAP2), astrocytes (GFAP), or microglia (Iba1) specifically revealed neuronal injury and glial reaction. In particular, more intensive MAP2 staining was observed in the striatum in the 2VO group compared to SHAM, indicative of the dynamic reorganization of axonal microtubules (Figures 3C–E). Reactive astrocytes labeled with GFAP were prominent in the frontal cortex ipsilateral to MCAO (ischemic hemisphere), especially with respect to the contralateral, non-ischemic hemisphere (Figures 3C–E). In the striatum, the GFAP signal was virtually lost in the hemisphere ipsilateral to MCAO, perceived as a sign of irreversible astrocyte injury (Figures 3C–E). The arborization index of Iba1-positive microglia was greatly decreased in the cortex and striatum ipsilateral to MCAO, clearly demonstrating microglia activation and neuroinflammation in focal cerebral ischemia (Figures 3C–E).

### Changes in the respiratory system induced by cerebral ischemia

**FIGURE 4:**
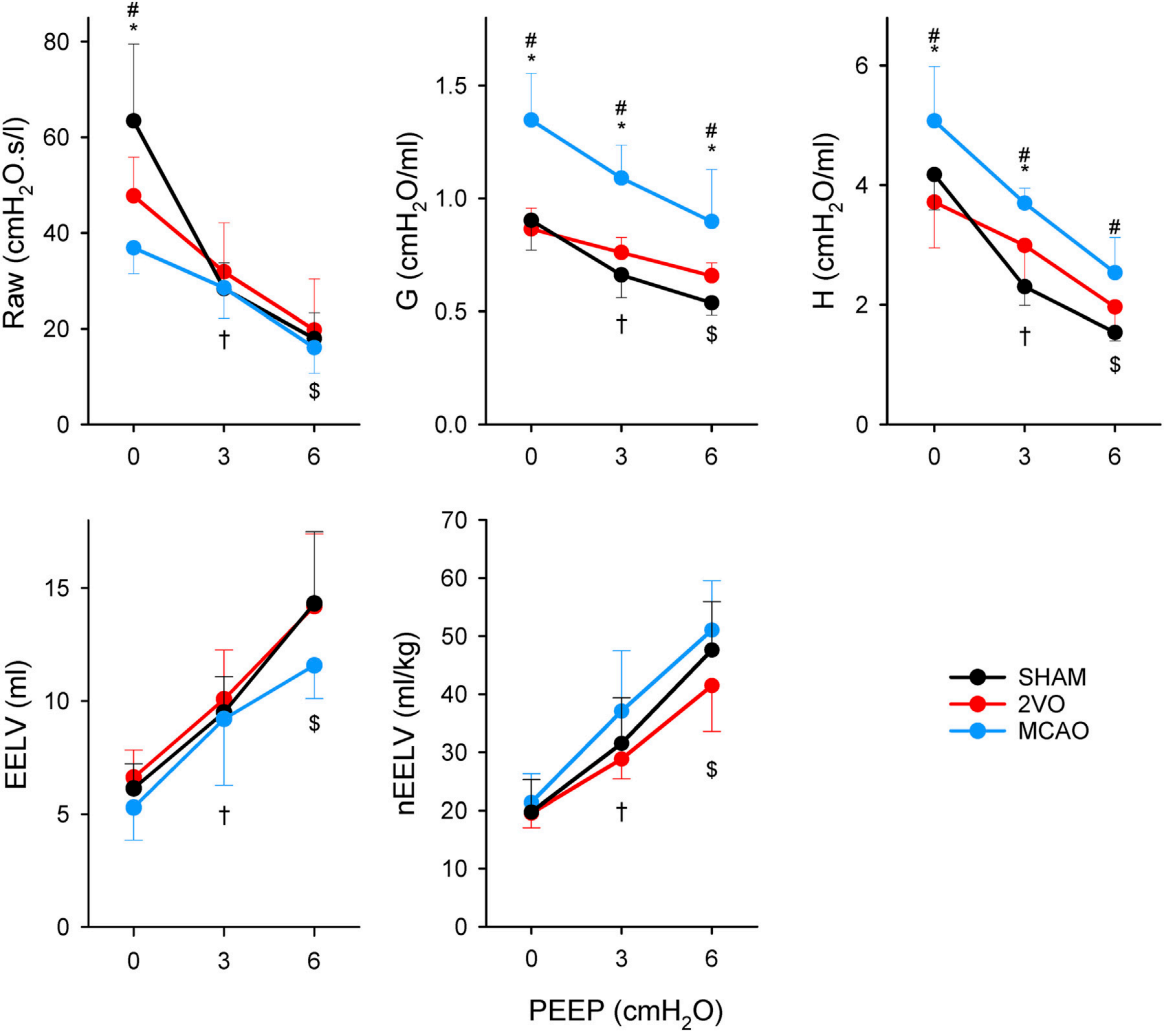
Airway resistance (Raw), tissue damping (G) and elastance (H) of respiratory tissues and end-expiratory lung volume (EELV) obtained at different levels of PEEP in sham-operated animals (SHAM, N = 7), and after bilateral occlusion of the common carotid artery (2VO, N = 7) and occlusion of the middle cerebral artery (MCAO, N = 7). *: p < 0.05 MCAO vs. 2VO; #: p < 0.05 MCAO vs. SHAM; †: p < 0.05 PEEP 3 vs. PEEP 0 in all groups; $: p < 0.05 PEEP 3 vs. PEEP 6 in all groups.

Figure 4 shows changes in respiratory mechanical parameters and in EELV in ventilated study groups at different levels of PEEP. There was a significant difference between the MCAO and SHAM groups at PEEP 0 in Raw (p = 0.04). Respiratory tissue mechanical parameters were significantly higher in the MCAO group compared to the other two groups at all levels of PEEP (p < 0.001 and p < 0.05 at all levels of PEEP for G, and H, respectively). No significant differences were detected in EELV and nEELV between the protocol groups. Elevating PEEP from 0 to 3 cmH_2_O and from 3 to 6 cmH_2_O caused significant decreases in respiratory mechanical parameters in all protocol groups, which were associated with significant increases in lung volume indices (p < 0.05 for all).

**FIGURE 5:**
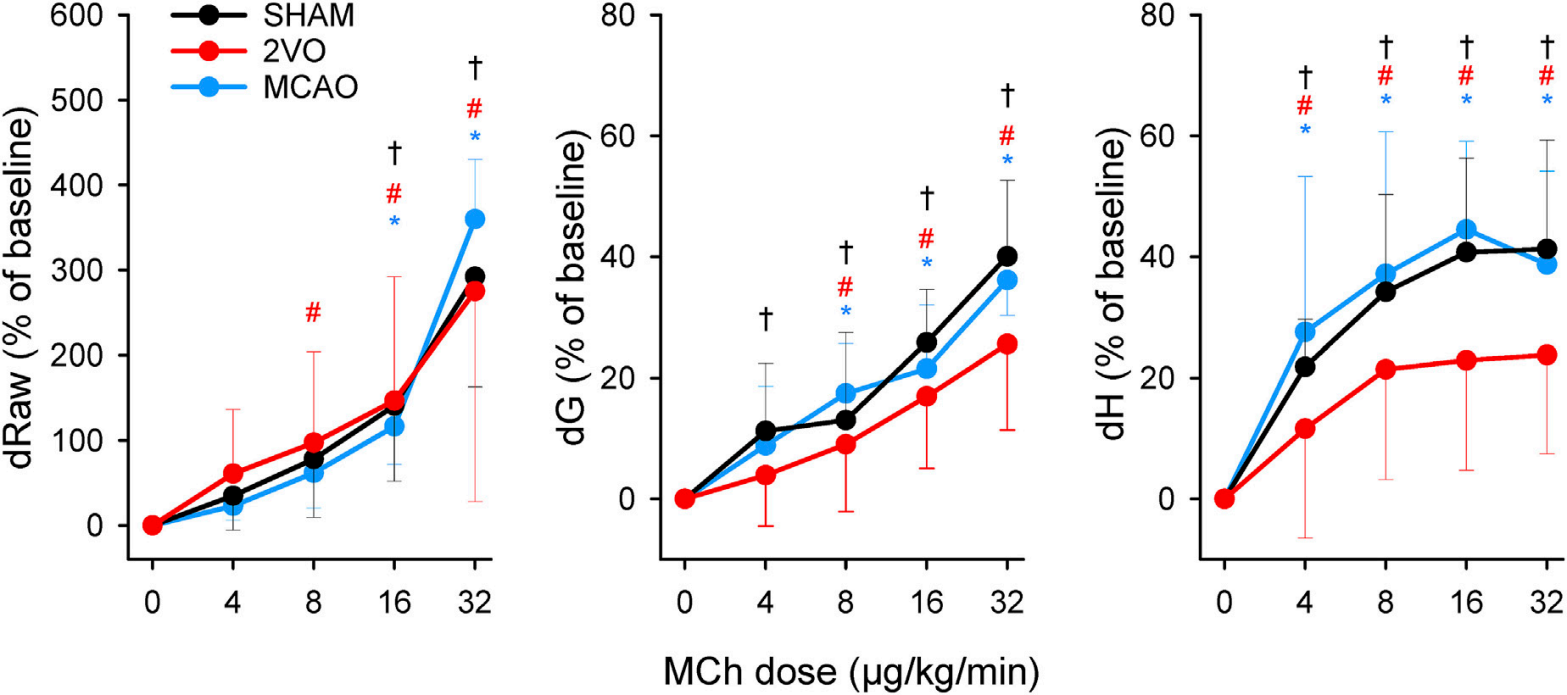
Changes in airway resistance (Raw), tissue damping (G), and elastance (H) during challenges with methacholine bronchoprovocation with increasing doses of the agonist (4–32 μg/kg/min) in the sham-operated animals (SHAM, N = 7), and after bilateral occlusion of the common carotid artery (2VO, N = 7) and middle cerebral artery occlusion (MCAO, N = 7). *, # and †: p < 0.05 vs. baseline (“0”) within the groups MCAO, 2VO and SHAM, respectively.

The results of the bronchoprovocation tests are summarized in Figure 5. Infusion of MCh caused dose-dependent increases in airway and respiratory tissue mechanical parameters (p < 0.001), with no significant difference between groups.

The lung edema index assessed from rats from the nonventilated groups when calculating the weight-corrected wet-dry ratio was significantly higher in the MCAO group than in the SHAM group (p < 0.05, Table 2). Lung histological analyses also confirmed the development of pulmonary edema around the pulmonary vessels, with the perivascular pulmonary edema index of 0.38 ± 0.33, 0.68 ± 0.85, and 1.45 ± 1.51 for groups SHAM, 2VO and MCAO, respectively (p < 0.02 between SHAM and MCAO groups).

**TABLE 2:** Assessment of pulmonary edema development by calculating lung tissue weight loss.

	SHAM	2VO	MCAO
Tissue weight loss (%)	76.2 (75.1–77.7)	78.0 (76.7–80.4)	77.3 (69.4–79.4)
Lung tissue weight loss corrected to body weight (%/g)	0.216 (0.201–0.268)	0.250 (0.222–0.271)	0.290* (0.243–0.354)

Lung tissue weight loss is expressed as percentage of wet weight and normalized to the body weight obtained before the measurements. Abbreviations: SHAM: sham surgery; 2VO: bilateral common carotid artery occlusion, MCAO: middle cerebral artery occlusion. *: p < 0.05 vs. SHAM and 2VO groups. Data are given as mean (max-min).

**FIGURE 6:**
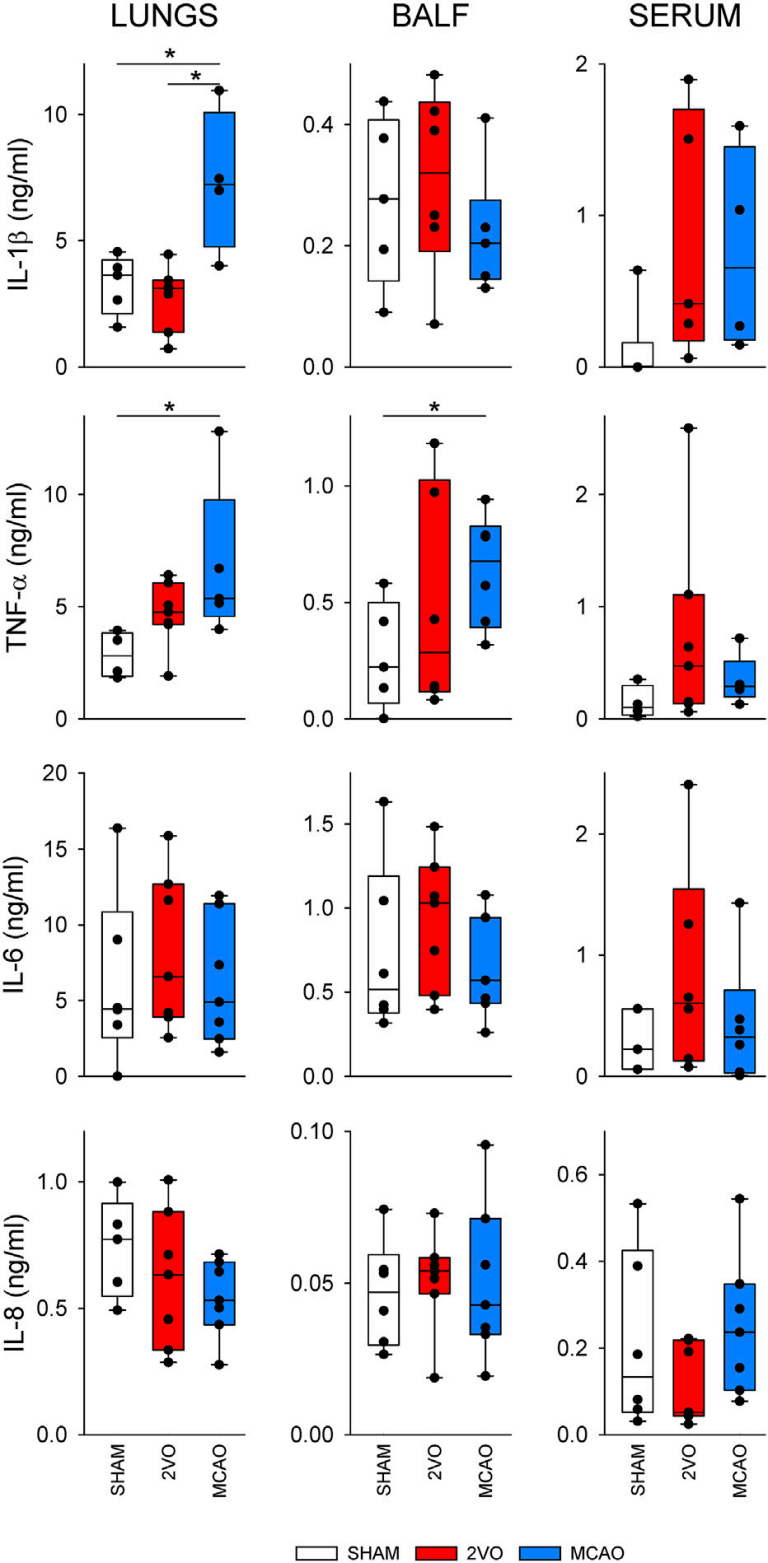
Inflammatory cytokine levels in lung tissue (left), bronchoalveolar lavage fluid (BALF, middle) and blood serum (right) obtained in sham-operated animals (SHAM, N = 6), and after bilateral occlusion of the common carotid artery (2VO, N = 7) and occlusion of the middle cerebral artery (MCAO, N = 7). IL-1β, interleukin-1β; TNF-α, tumor necrosis factor α; IL-6, interleukin-6; IL-8, interleukin-8. *: p < 0.05.

Figure 6 shows cytokine levels in lung tissue homogenate, BALF, and blood serum samples obtained from the nonventilated group. In lung tissue homogenates, IL-1β levels were significantly elevated in the MCAO group compared to the SHAM and 2VO groups (p < 0.05 for both) and increases in TNF-α were observed compared to the SHAM group (p < 0.05). This elevation of TNF-α was also observed in BALF (p < 0.05). No significant differences were observed in serum cytokine levels and we found no difference in IL-6 and IL-8 levels between the protocol groups.

There was no detectable amount of total bile acids in the BALF in any non-ventilated experimental group.

## Discussion

The present experimental study revealed that deteriorations in lung tissue mechanical properties and pulmonary edema develop 3 days after focal brain ischemia induced by permanent unilateral occlusion of the middle cerebral artery. These adverse respiratory tissue changes were not reflected in the airway properties and the end-expiratory static lung volume. Conversely, permanent global cerebral ischemia did not cause detectable respiratory mechanical changes. Neither focal nor global cerebral ischemia affected lung responsiveness to exogenous cholinergic constrictor stimuli. Focal ischemia resulted in significant loss of body mass, associated with elevated levels of hematocrit and hemoglobin. Although focal cerebral ischemia did not affect PEEP-dependent changes in respiratory mechanical parameters and static lung volume, increasing PEEP did not normalize increased respiratory tissue damping and elastance.

To validate the presence of cerebral ischemia, TTC staining and fluorescent immunohistochemistry were used on brain slices and sections. These results confirm the validity of the cerebral ischemia models adopted in the present study to mimic the main features of human chronic hypoperfusion (2VO) (Farkas et al., 2007; Farkas and Luiten, 2001) and acute focal cerebral ischemia (MCAO) (Lopez and Vemuganti, 2018). The 2VO intervention allows limited compensation for hypoperfusion by the vertebral arteries through the circle of Willis, thereby explaining the lack of cerebral infarction. Conversely, MCAO occludes a terminal artery; thus, the subsequent regional ischemia cannot be compensated. Microglia and astrocytes interact dynamically during ischemic injury, influencing both neuronal damage and repair. Damage-associated molecular patterns at the injury site activate microglia (Patabendige et al., 2021), which polarize into pro-inflammatory (M1) or anti-inflammatory (M2) phenotypes. M1 microglia release cytokines like IL-1β, IL-6, and TNF-α, promoting inflammation, while M2 microglia secrete factors like IL-10 and vascular endothelial growth factor to support tissue repair, neurogenesis and angiogenesis. Reactive astrocytes, activated by these cytokines, form protective glial scars and contribute to neuroprotection through glutamate uptake, though they can also amplify inflammation by releasing cytokines, ATP, and glutamate (Liang et al., 2023).

Significant loss of body mass was observed in MCAO rats 3 days after the intervention, and loss of body mass in the 2VO group was also significant, although markedly smaller. Since MCAO has been shown to cause hemiplegia/hemiparesis with subsequent swallowing difficulties (Cullins et al., 2021), this dysfunction may explain our findings. Interestingly, weight loss was not reflected in EELV or nEELV alterations, suggesting a maintained chest configuration after each intervention. This finding suggests that the contribution of chest wall mechanics to the respiratory mechanical parameters was not affected by the induction of cerebral ischemia, and our findings reflect the differences between the pulmonary manifestations of the focal and global brain injury.

Separate assessment of the mechanical parameters of the airway and respiratory tissue revealed fundamentally different alterations in these compartments from cerebral ischemia. MCAO resulted in significantly reduced Raw at low PEEP level (0 cmH_2_O). This finding may be explained by the increased sympathetic activity after cerebral ischemia (Meisel et al., 2005; Prass et al., 2003), which subsequently diminishes bronchial smooth muscle tone, leading to airway dilation (Tank and Lee Wong, 2015). Elevating PEEP as an increased mechanical load may overcome this phenomenon, resulting in no difference in airway mechanical properties. This finding agrees with previous findings that demonstrate normal airway resistance assessed by the end-inspiratory occlusion method 24 h after focal ischemia (Samary et al., 2018), and suggests that the reduced Raw may be due to airway-parenchymal interdependence (Bates and Rajendran, 2018). In contrast, MCAO caused significant deterioration in tissue parameters, as represented by elevations in both dissipative (G) and elastic (H) properties of respiratory tissues at all levels of PEEP. The development of pulmonary edema demonstrated by the more pronounced loss of weight in lung tissue (Table 2.) may be responsible for this effect. This phenomenon was apparent in earlier investigations demonstrating neurogenic pulmonary edema in experimentally induced cerebral ischemia (Toung et al., 2005; Wong et al., 2011) and in stroke patients (Verein et al., 2010). Furthermore, in agreement with previous findings (Naseh et al., 2020; He et al., 2016; Offner et al., 2006), overexpression of inflammatory cytokines was also found in lung tissue and BALF (Figure 5). This pulmonary inflammatory disorder can play a role in the development of edema and can also lead to increased tissue resistance and elastance through structural changes in lung tissue and/or peripheral airways (Dellaca et al., 2008; Rassler, 2007). Hemoconcentration developed after MCAO can further aggravate these detrimental effects, as elevated hematocrit was shown to increase G and H (Petak et al., 2016). Furthermore, unbalanced autonomic system may have also contributed to the altered respiratory tissue viscoelasticity (Yang et al., 2022). To assess whether the possible presence of aspiration is a contributing factor to the adverse lung tissue mechanics (Fodor et al., 2014), we measured total bile acid levels from the BALF. There was no evidence for an elevated bile acid level in the BALF in the animals with brain ischemia. This suggests that aspiration did not contribute to lung inflammation causing increases in G and H. It is important to note that both G and H depend on functional lung volume. Although the animals in the MCAO group lost a significant body mass, the EELV did not differ significantly between the MCAO and other groups. This finding confirms the primary role of mechanical changes in intrinsic lung tissue without involvement of alveolar derecruitment.

Despite the considerable impact of stroke on the pulmonary system, reports on the effects of cerebral ischemia on respiratory mechanics are scarce. Unlike our results, no change in lung elastance was reported 24 h after focal ischemia in rats (Samary et al., 2018). The difference between the 24-h and 3-day time window after ischemia may explain this discrepancy, indicating that deterioration in the mechanics of the respiratory tissue requires more time to develop.

It should be noted that deteriorations in the mechanical properties of the respiratory tissue were not reflected in the blood gas parameters (Table 1). This seemingly controversial finding in the MCAO group can be explained by maintained V/Q ratio despite the compromised tissue mechanics.

While the application of high PEEP in mechanically ventilated patients is often considered to improve gas exchange, this adjustment requires particular attention in the presence of stroke due to the potential for intracranial hypertension to develop (Robba et al., 2019; Mrozek et al., 2015). In our study, the increase in PEEP did not normalize the mechanics of respiratory tissues. This finding may indicate the possibility of higher ventilation pressures developing in the respiratory system to an elevated PEEP after cerebral ischemia, thus increasing the risk of barotrauma in the lungs. Accordingly, our results may have an impact on the optimization of ventilation settings in the respiratory management of stroke patients.

Neural control plays a major role in the regulation of airway smooth muscle tone. While 2VO or MCAO was likely to disturb this regulation, as indicated by the lower Raw in the latter group, airway responsiveness to MCh was not affected by focal or global cerebral ischemia. The lack of modulation of airway responsiveness may be related to the development of ischemic injury primarily in the forebrain after both 2VO and MCAO, while these interventions had no significant detrimental consequences on the breathing centers located in the medulla oblongata. To our knowledge, there has been only one study assessing alterations in airway hyperresponsiveness after stroke. In contrast to our findings, the development of airway hyperresponsiveness was demonstrated in a focal ischemia thermocoagulation model 24 h after intervention (Samary et al., 2018). The difference between these earlier results and the present findings can be explained by the more pronounced expression of pro-inflammatory circulating cytokines in their study.

Regarding the inflammatory profile after global and focal ischemia, we found significant elevations in TNF-α in lung tissue after MCAO, in agreement with previous studies using similar models in a comparable time window (Naseh et al., 2020; Samary et al., 2018; He et al., 2016). Changes in this inflammatory cytokine in the serum are more controversial, with some studies reporting elevations (Su et al., 2022; Liesz et al., 2009), while in agreement with the present results, others demonstrated a lack of change after an MCAO insult (Prass et al., 2003; Austin et al., 2019). We found no earlier report demonstrating concomitant elevations in the IL-1β levels in lung tissue seen in the present study after MCAO. However, similar to our results, the serum level of this inflammatory cytokine did not change after focal cerebral ischemia (Austin et al., 2019). The reason for inconsistent inflammatory results may be due to differences between species or between strains (Campos-Martorell et al., 2014), and/or differences in the experimental models used to induce cerebral ischemia, i.e., permanent or transient occlusion of the middle cerebral artery. In the event of permanent occlusion, suppressed systemic inflammation is associated with local brain inflammation, leading to long-lasting post-stroke immunodepression in the periphery (Shim and Wong, 2016; Liu et al., 2018; Chamorro et al., 2007; Santos Samary et al., 2016; Dirnagl et al., 2007; Winklewski et al., 2014).

Some limitations related to the current study warrant consideration. We used young, healthy animals with no initial inflammation or comorbidity, which is common in experimental models, but atypical for stroke patients. Earlier experimental studies using transient MCAO revealed that age is positively correlated with the severity of post-stroke lung infections (Zhang et al., 2021) and negatively related to neutrophil function (Wen et al., 2022). Accordingly, our findings are expected to manifest in a more severe form in aged subjects. In addition, animals lost following MCAO may have had the most severe respiratory abnormalities. Another limitation is related to the use of only male animals, while 55% of ischemic stroke occurs in women (Feigin et al., 2022). Further experiments are required to reveal potential sex-related differences in brain-lung interactions. In the present study, we adapted an experimental model that mimics permanent global or focal cerebral ischemia. This model has translational value for the vast majority of cases of ischemic stroke where recanalization of the occluded vessel is not performed due to a longer time window or due to not meeting therapeutic requirements (French et al., 2016). The current study does not allow a separate assessment of the potential role of dehydration in the altered respiratory mechanical changes following ischemic stroke; future studies involving animals with invasive fluid supplementation are required to clarify this effect on the respiratory consequences.

In conclusion, an experimental model of focal cerebral ischemia induced by permanent occlusion of the middle cerebral artery for 3 days decreases airway resistance at low lung volumes and deteriorates respiratory tissue damping and elastance. Elevating PEEP levels during mechanical ventilation was not effective in normalizing impaired respiratory tissue mechanics. Global cerebral ischemia induced by permanent occlusion of both common carotid arteries did not alter airway and respiratory tissue mechanics. The impaired tissue mechanics was associated with a lack of change in end-expiratory lung volume, the development of pulmonary edema and elevations in pro-inflammatory cytokine levels in lung tissue after focal cerebral ischemia, indicating the primary role of intrinsic changes in lung tissue mechanics without involvement of alveolar derecruitment. Since the characterization of respiratory mechanical changes after stroke is of great importance to guide mechanical ventilation strategy, our findings can contribute to optimizing this lifesaving modality in this substantial public health burden.

## Data Availability

The raw data supporting the conclusions of this article will be made available by the authors, without undue reservation.
